# Nitrogen fertilization and precipitation affected Wheat (*Triticum aestivum* L.) in dryland the Loess Plateau of South Shanxi, China

**DOI:** 10.1016/j.heliyon.2023.e18177

**Published:** 2023-07-13

**Authors:** Hafeez Noor, Fida Noor, Li Ting Liang, Pengcheng Ding, Min Sun, Zhiqiang Gao

**Affiliations:** a Collage of Resources and Environment, Shanxi Agricultural University, Taigu 030801, China; b College of Agriculture, Shanxi Agricultural University, Taigu, 030801, Shanxi, China; c College of Veterinary Medicine, Shanxi Agriculture University, Taigu 03080, Shanxi, China

**Keywords:** Dryland, Nitrogen fertilizer, Precipitation, Winter wheat, Water consumption

## Abstract

Wheat (*Triticum aestivum* L.) is a staple crop worldwide, and its yield has improved since the green revolution, which was attributed to chemical nitrogen (N) fertilizer application. An experiment was conducted to set seven nitrogen application levels of N0, N90, N120, N150, N180, N210 and N240 kg ha^−1^ before sowing. The results showed that grain yield under the nitrogen rate of N210 kg ha^−1^ was significantly increase the water intake during jointing to anthesis, Soil water storage of dryland wheat in fallow period was higher than water consumption in jointing stage and the leaf area index at anthesis, the tiller percentage rate, the jointing-anthesis, and nitrogen accumulation were closely related to yield and its components. Nitrogen fertiliser rate N150 kg ha^−1^ significantly increased dry matter buildup from jointing to flowering in dryland wheat compared to N fertiliser rate N210 kg ha^−1^. The rise of nitrogen application rate, there were no significant variance in nitrogen accumulation of Stem + leaf sheath and cob + glume at maturity, respectively. N fertiliser rate N210 kg ha^−1^ compared to N180 kg ha^−1^ significantly reduced grain gliadin content in dryland wheat, respectively. Wheat crops under N210 kg ha^−1^ could achieve both high NUE and grain yield simultaneously with only moderate N fertilizer in South Shanxi, China.

## Highlights

• N input was optimized to match year type based on fallow season precipitation.
• Soil water storage in 80–300 cm depth was important for yield formation.
• Optimized N input regulated water consumption during wheat development.
• Optimizing N input for dryland wheat production was feasible and economical.

## 1 Introduction

China is the largest producer of wheat in all over world. The North China Plain is fertile, and it is one of the most densely populated regions in the world. The plain is one of China's most important agricultural regions, producing wheat, in China, accounting for 25% of national food production [1]. The Loess Plateau which is one of the key wheat (*Triticum aestivum* L.), generating zones in China, and its yield regulates food security. System Fertilization is a new fertilization concept that relies on biological nutrient cycling between rotation phases to achieve nutrient-use efficiency and thereby reduce mineral nutrient input requirements for winter wheat growth and the sustainable development of cultivated land resources [2]. In numerous parts of the world, a great increase in N fertilizer input was essential to increase crop profit [3]. Natural precipitation is the only water basis for wheat growth in dryland, which determines the soil water status. Soil water shortfall affects the growth, and progress of wheat in each key growth stage, negatively affects water use of wheat, and plant dry matter accumulation, and leads to the decline of wheat yield and grain quality [4,5]. The shortage of water in the critical growth stage of wheat is usually caused by the deficiency of soil water standby due to inadequate rainfall in the early stage or excessive ingesting of soil water, resulting in the decline of wheat yield [6].

Many studies on drought treatment in wheat sowing stage and wintering stage showed that the plant biomass of wheat significant descending trend, and re-watering in later stage could not compensate for the biomass loss caused by drought [7]. During fallow period determines soil moisture level before sowing in dryland wheat fields. Sufficient precipitation before sowing is conducive to the smooth emergence of dryland wheat. Meanwhile, wheat can make sustainable use of soil moisture stored before sowing to the period of elongation, will seriously affect the appearance rate of wheat [8]. It has been believed that during fallow period in dryland wheat fields rainfall played an important role in promoting tillering, regreening, jointing and establishing excellent populations, and healthy individuals in dryland wheat [9]. Rational application of nitrogen fertilizer not only supplements soil nutrients, but also promotes soil water-nitrogen interaction, and promotes crop growth [10]. The nitrogen application reduced soil water consumption before jointing in dryland wheat, provided sufficient soil water for later growth, and development of wheat, improved water use efficiency, spike number per area, and thus increased yield. Water intake in dryland wheat growing season is far greater than precipitation. Long-term excessive nitrogen application will cause excessive consumption of soil reservoir in wheat growing season, and it is difficult to maintain stable soil reservoir for a long time, limiting the improvement of water use efficiency and yield [11]. The excessive nitrogen application would make the soil water consumption of dryland wheat too high before jointing, and lead to yield reduction. How to apply fertilizer rationally in combination with precipitation characteristics in dryland wheat areas to improve wheat water use efficiency and yield is an urgent production problem to be solved [12,13]. N was used to predict the effects of different precipitation gradients on the yield of dryland wheat under optimized cultivation measures, so as to provide scientific and reasonable field cultivation measures wheat production, to achieve high yield and efficient production of dryland wheat. The main objectives of this study were to (1) clarify the effects of N application rate under N210 kg ha^−1^ on population development and yield formation, and (2) determine whether N210 kg ha^−1^ could help improve both yield and NUE simultaneously.

## 2 Materials and methods

**Fig. 1:**
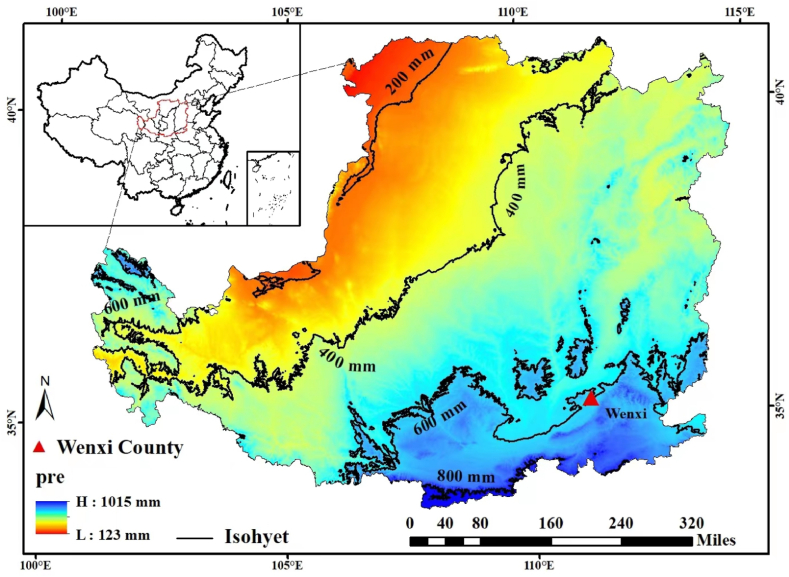
Location of experiment site in the Loess Plateau. The regional distribution of an-nual precipitation is shown in different colors on the map [6]. (For interpretation of the references to color in this figure legend, the reader is referred to the Web version of this article.)

Field experiments were conducted in a farmer field in wenxi, shanxi province (35° 24 N, 111° 26 E). The main soil types were loam, pH 8.2, organic matter 9.83 g kg^−1^, total nitrogen 0.82 g kg^−1^, ammonium nitrogen 2.4 mg kg^−1^, nitrate nitrogen 11.1 mg kg^−1^, available phosphorus 15.9 mg kg^−1^. Available potassium 133.0 mg kg^−1^. According to the classification technique of rain year varieties in fallow period, the precipitation in fallow period was fewer than 220.7 mm, the precipitation in fallow period was 220.7–437.0 mm, and the precipitation in fallow period was supplementary than 437.0 mm, and the precipitation in fallow period was wet. The precipitation of 2009–2010, 2012–2013, 2015–2016 and 2016–2017 were 149.1 mm, 205.1 mm, 126.8 mm and 153.3 mm, respectively which were perennial Figure (1).

### 2.1 Experimental design

The wheat variety ‘‘Yunhan-20410″ was providing by Agriculture and Rural Bureau of wenxi. A randomized complete block design was used to set seven nitrogen application rate N0, N90, N120, N150, N180, N210 and N240 kg ha^−1^ before sowing. The plot area was 10 m × 6 m = 60 m^2^. From three repetitions where. The conventional nitrogen application rate of farmers was N210 kg ha^−1,^ average nitrogen application rate in the survey area was 205 kg ha^−1^, and 0 kg ha^−1^.

**Fig. 2:**
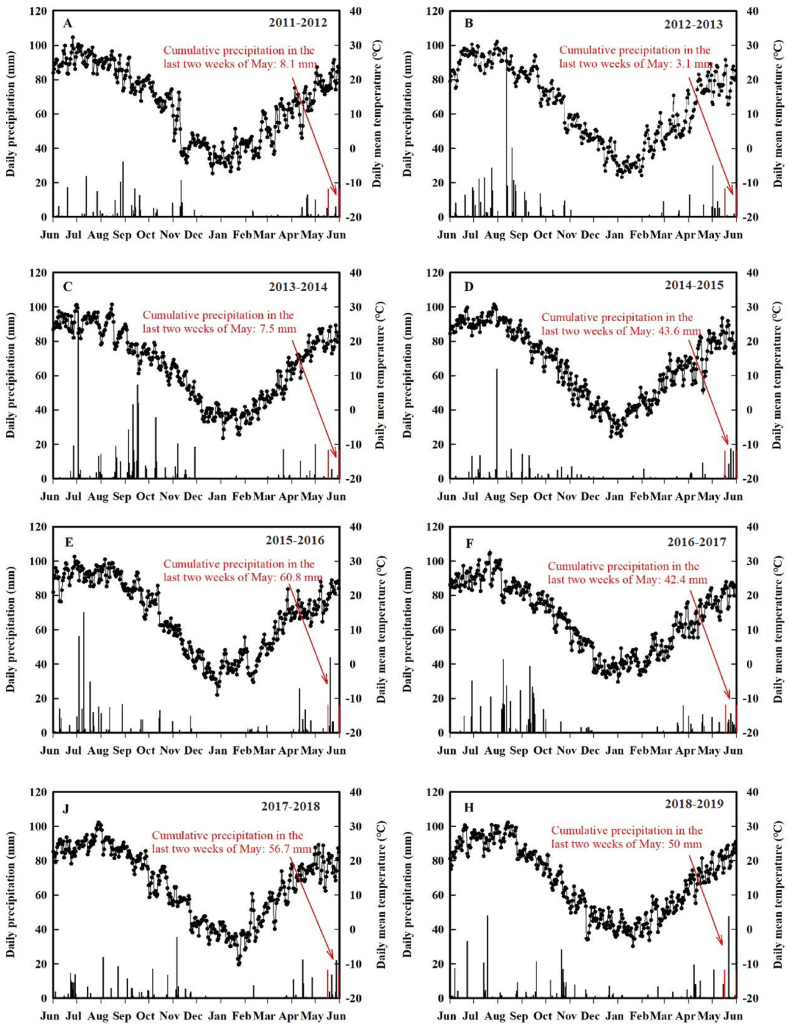
Daily precipitation and average temperatureon Shanxi Wenxi in 2011–2019.

**Fig. 3:**
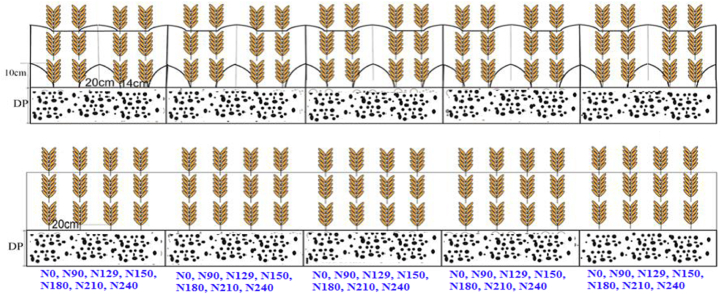
Illustration of different Nitrogen fertilizer in dryland the Loess Plateau of South Shanxi.

In previous winter wheat crop which were harvested with 20–30 cm of high stubble was carried out in early and middle July, and the land was leveled with shallow rotation at the end of August. The seeds were sown at the end of September or early October with 60 kg P_2_O_5_ ha^−1^, and 30 kg K_2_O ha^−1^ as phosphate fertilizer, and potassium fertilizer before sowing for detailed (Fig. 2). For detailed field management (Table 1) (see Fig. 3).

**Table 1:** Information on land preparation, field management, and operation procedures in the experiment.

Date of tillage, sowing harvest	Experiment years							
	2011–2012	2012–2013	2013–2014	2014–2015	2015–2016	2016–2017	2017–2018	2018–2019
Date of deep ploughing	15 Jul	14 Jul	9 Jul	14 Jul	16 Jul	16 Jul	8 Jul	11 Jul
Date of sowing	29 Sep	28 Sep	29 Sep	28 Sep	2 Oct	2 Oct	28 Sep	2 Oct
Date of harvest	30 May	28 May	2 Jun	29 May	2 Jun	5 Jun	4 Jun	28 May
Operation procedures								
N0, N90, N120, N150, N180, N210, N240	Previous harvesting→ 20–30 cm Tall stubble (20−30 cm) was left→ Deep ploughing once in early or middle July after previous harvesting→ Rotary tillage once before sowing→ Fertilization and Sowing→ Harvest							

### 2.2 Soil moisture

Before sowing the soil samples from 0 to 300 cm (one soil layer per 20 cm) were taken, A flat plot was selected, a 3 m deep section was dug, samples were taken every 20 cm layer, and the ring knife technique was used to measure the soil bulk density. The ring knife technique is a method used to measure soil bulk density. It involves using a cylindrical metal ring that is driven into the soil to a known depth using a hammer or mallet. The soil inside the ring is then removed and weighed, and the volume of the ring is calculated. The bulk density of the soil is then calculated by dividing the dry weight of the soil by the volume of the ring.

### 2.3 Soil basic nutrients

In the beginning of sowing maturity stage the soil samples of 0–20 cm soil layer were taken, and screened after air drying to determine the contents of soil carbon-based, available phosphorus, alkali-hydrolyzed nitrogen and potassium.

Soil organic carbon: after drying, soil samples were screened by 0.25 mm, and the total soil organic carbon content was determined by potassium dichromatic volumetric method.

Soil alkali-hydrolyzed nitrogen: after air drying, soil tasters were partitioned by 1 mm sieve, and the contented of alkali-hydrolyzed nitrogen was determine by alkali-hydrolyzed diffusion technique.

Soil available P and K: the soil samples were dried and screened by 1 mm, and the contents of available P, and K were resolute by 0.5 mol L^−1^ NaHCO_3_ extraction-Molybdenum-antimony resistance. Colorimetric analysis is a technique that uses colored compounds to measure the concentration of a substance in a solution, in this technique 1 mol L^−1^ NH_4_OAC extraction-flame spectrophotometry, respectively.

### 2.4 Soil bulk density (BD), soil water storage (SWS) and soil water consumption (SWC)

Soil bulk density, water storage and water consumption were calculated by referring to Ref. [6].

Soil bulk density (BD):

$$Soil\;bulk\;density:D=\left(M_{1}-M_{0}\right)/V$$

Where, D is soil bulk density (g/cm3); M1 is the mass of soil and ring cutter after drying (g); M0 is the mass of ring cutter (g); V is the ring cutter volume (cm^3^).

Soil water storage (SWS):

SWSi=WI×Di×Hi×10/100

Where, SWS stands for soil water storage (mm); W stands for soil water content (%); D represents soil bulk density (g cm^−3^); H is the soil layer thickness (cm), and the soil layer.

Soil Water consumption (SWC):

ET=ΔS+P

Where, ET is water consumption during growth period (mm); ΔS is the decrease of soil water storage in a certain growth stage (mm). *P* is effective precipitation (mm) during the growth period.

### 2.5 Agronomic traits and dry matter accumulation

Population dynamics: Three rows of wheat plants at 0.667 m^2^ were selected at a fixed point stage of wintering, jointing, flowering, booting and maturity to investigate population dynamics. Leaf area index (LAI) According to the method of 10 plants were selected from each plot in each growth stage to measure the width, length and the inverted second leaf, and calculate the leaf area. Leaf area (cm2) = length × width × 0.73 × number in green leaves.

### 2.6 Dry matter quality

Taken 20 strains of plant growth representative, and were divided into two groups, roots and cut out the over-wintering stage (the plant), jointing stage (leaf and stem), flowering stage (leaf, ear, stem and leaf sheath), adulthood (leaf sheath, leaf, stem, cob + glume shell and grain), each part were respectively on annotation processing kraft paper bag, First 105 °C for 1 h, then adjust the oven to 70 °C, bake to continuous weight. The sample were weighed and crushed.

### 2.7 Yield and yield components

The effective spike number of 0.667 m^2^ evenly growing wheat sample segment was investigated in each plot by removing side rows at maturity stage. Then 20 spike were arbitrarily selected from individually plot, and the average grain number per spike was calculated after drying. Five groups of grain samples (1000 grains per group) were randomly selected, and the 1000-grain weight was determined by the models. 20 m^2^ was randomly selected from each treatment for yield measurement. Grain moisture content was measured by grain moisture meter (KETT PM-8188-A, Japan), and the national grain storage standard water content is 13%. This means that the moisture content of grain should not exceed 13% when it is stored. Reporting crop yield at a standardized moisture content is important for proper grain storage, standardization across buying points, and the comparison of results across research trials. The accumulation, partitioning, and translocation of dry matter and N were calculated using the following equations [14,15].

[1] Post-anthesis dry matter production (DM _post_, kg ha^−1^) = Total dry weight at maturity – TDW_as_

[2] Harvest index (%) = Grain dry weight/Total dry weight at maturity

Where TDW_as_ (kg ha^−1^) is total dry weight at anthesis. TN (kg ha^−1^, and TN_as_ (kg ha^−1^) are total N quantity at maturity and anthesis, respectively. GN (kg ha^−1^) is grain N content.

Where, Y_N_ and Y_0_ are yield (kg ha^−1^) in N fertilization and N0 treatment, respectively. TN_N_ and TN_0_ are total N quantity at maturity (kg ha^−1^) in N fertilization and N0 treatment, respectively. N_F_ is the total input of fertilizer N (kg ha^−1^).

### 2.8 Nitrogen content in plants

The crushed plant samples were boiled with H_2_SO_4_–H_2_O_2_, and cooled to a constant volume of 50 mL, diluted by 10 times, 1 mL of diluent was taken, 1 mL of EDTA-methyl red solution was dropped, and tit-rated with 0.6 N NaOH solution. After the solution turned from red to yellow, 5 mL sodium hypochlorite solution, and 5 mL phenol solution were successively added, shaken, and distilled water was constant volume to 50 mL. After 1 h determined with a calorimeter of 1 cm light diameter, and the blank dissolving solution with the same dissolving solution, and various reagents was zeroed. Twenty (20) ears of wheat with uniform growth were picked in the sample at the mature stage, the grains were stripped, and placed in the oven to dry at 70 °C. The dried grains were crushed by a disc experimental grinding mill (Perten, Sweden) for the determination of protein content. The nitrogen content of grains was determined by h_2_SO_4_–H_2_O_2_-indiophenol blue colorimetric method and the protein content was multiplied by 5.7. Each sample was repeated three times. Incessant extraction technique was used to regulate the protein components of grains.

### 2.9 Statistical analysis

Values are presented as mean ± SD. The experiment used Microsoft Excel 2010 for data sorting and graphing, and SPSS-22 software for one-way ANOVA and statistical analysis, LSD postmortem test was used to compare the differences between tillage methods, and the significance level was set at ɑ = 0.05.

## 3 Results

### 3.1 Soil water consumption in dryland wheat

**Fig. 4:**
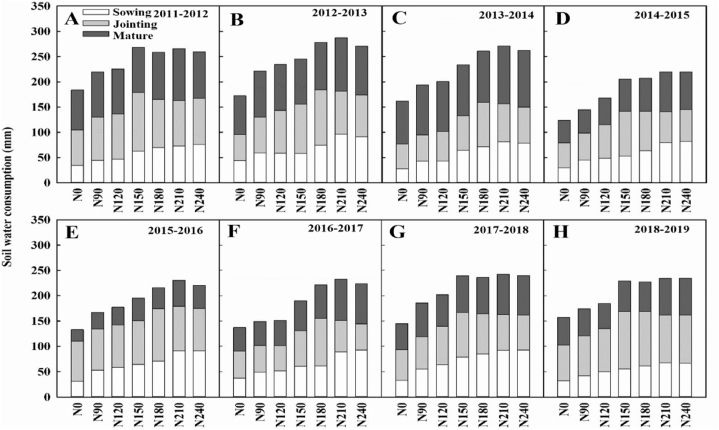
Effects of Nitrogen fertilizer on soil water consumption of different growth stages at 0–300 cm depth. (Upper-case letters and lower-case letters indicate comparisons between N rates between. letter means significant at 0.05 probability levels. Bars represent the standard error).

Soil water storing in the fallow period dryland wheat was higher than water consumption in the sowing to jointing stage Figure [4 (A, D, G, H)]. Perennial, soil water storage in 2010–2011 fallow period was higher than that in sowing flowering stage Figure [4 (b)], and soil water consumption in 2013–2014 fallow period was higher than that in sowing to flowering period under N fertiliser rate were 0, 90, 120, and 150 kg ha^−1^ Figure [4 (e)]. Soil water storage of dryland wheat in fallow period was higher than water consumption in jointing stage Figure [4 (C, F)]. That soil water consumption can supply water to wheat jointing stage in the fallow period, but can supply water to wheat flowering stage all the year round.

### 3.2 Effects of N fertiliser rate on tiller number and leaf area index in dryland wheat

The number of tiller in wheat populations at jointing stage was significantly reduced 12.1% at 150 kg ha^−1^ compared to N fertiliser rate 210 kg ha^−1^ in dryland. The tiller yield number was increased to 20.3%, respectively there was no significant change in tiller number of wheat population at jointing stage with the rise of nitrogen fertiliser. N fertiliser rate 210 kg ha^−1^, 180 kg ha^−1^, N fertiliser rate was significantly increased tiller numbers 6.6% (Table 2). The N fertiliser rate of 150 kg ha^−1^ and N180 kg ha^−1^ was more beneficial to tiller polarization, increase tiller ear rate, and lay the foundation for the formation of spike number in dryland wheat.

**Table 2:** Effects of N rate to tiller number at jointing and percentage of ear bearing tiller increase.

Year	Tiller number per hectare at jointing ( × 10^4^ ha^−1^)							Percentage of earbearing tillerincreas PET (%)						
	N0	N90	N120	N150	N180	N210	N240	N0	N90	N120	N150	N180	N210	N240
2011–2012	1104 b	1161 ab	1195 ab	1178 ab	1199 ab	1296 a	1329 a	35.52 b	35.86 b	37.5 ab	41.33 a	39.84 a	35.14 b	34.53 b
2012–2013	1332 c	1375 c	1482 b	1496 b	1556 a	1523 ab	1592 a	40.73 a	41.37 a	42 a	42.24 a	42.41a	41.25 a	39.64 a
2013–2014	1380 e	1364 e	1510 d	1585 c	1548 c	1774 a	1635 b	38.44 d	42.17 b	40.16 c	40.43 c	44.52 a	38.24 c	41.12 bc
2014–2015	1000 a	9426 b	990 a	1010 a	1042 a	1013 a	1028 a	42.3 c	46.67 b	47.35 a	48.26 a	45.46 b	44.65 bc	43.93 c
2015–2016	1091 e	1252 d	1401 c	1476 b	1444 b	1425 b	1629 a	37.25 ab	38.84 a	37.35 ab	37.45 ab	39.44 a	36.76 b	32.33 c
2016–2017	1029 ab	1168 a	1069 ab	986 b	1065 ab	1000 ab	1068 ab	42.62 d	42.67 d	47 c	52.94 ab	53.94 a	52.63 ab	49.74 b
2017–2018	891 d	986 c	1072 b	1069 b	1183 ab	1250 a	1301 a	42.94 c	43.87 c	45 b	47.44 a	42.24 c	37.56 d	36.23 d
2018–2019	961 d	983 d	1063 c	1042 c	1206 b	1261 a	1295 a	45.25 c	50.16 b	49.52 b	53.33 a	45.53 c	40.98 d	40.11 d
Mean	1092 c	1146 c	1209 b	1209 b	1271 ab	1300 a	1344 a	40.67 d	42.55 c	43.24 b	45.363 a	44.17 b	40.85 d	39.68 e
Y	*							*						
N	**							**						
Y*N	*							**						

Note: PET; percentage of earbearing tillerincreas. Different lowercase letters within a column and different capital letters within a row or column represent significant differences (*P* < 0.05).Means and standard errors of three replicates are presented. The means are not significantly different within a given season when followed by the same lowercase letter using LSD at *P* < 0.05. “**” indicates significant difference between the two optimal N rates in dry and normal year, “ns” indicates no significant difference. The same below.

The leaf area index (LAI) of wheat at jointing stage was significantly decreased by 15.6% at the nitrogen application rate of N150 kg ha^−1^ compared to N210 kg ha^−1^ where increased during anthesis during increasing of nitrogen application rate leaf area index did not change significantly, which was about 3.39–3.54%. The leaf area index at jointing stage was significantly decreased by 20.5% at N180 kg ha^−1^ compared to N210 kg ha^−1^. At flowering stage increasing of nitrogen application rate, leaf area index was non-significant, about 3.67–3.86% (Table 3). N150 kg ha^−1^ in drought year and N180 kg ha^−1^ in all year was beneficial to increase jointing to flowering leaf area index of dryland wheat.

**Table 3:** Effects of N rate on LAI at jointing and anthesis stage in dryland wheat.

Year	Leaf area index at jointiong (LAI_J_)							Leaf area index at anthesis (LAI_A_)						
	N0	N90	N120	N150	N180	N210	N240	N0	N90	N120	N150	N180	N210	N240
2011–2012	1.24 d	1.45 c	1.54bc	1.65 b	1.89 a	1.93 a	1.96 a	2.65 e	3.33 d	3.41 c	3.49 c	3.25 d	3.92 a	3.66 b
2012–2013	1.48 d	1.78 c	1.81 c	1.81 c	1.94 c	2.26 b	2.35 a	3.13 c	3.61 b	3.66 ab	3.78 ab	3.84 a	3.82 a	3.83 a
2013–2014	1.88 c	1.89 c	1.94 c	1.91 c	1.99 c	2.51 a	2.25 b	3.22 b	3.6 ab	3.63 ab	3.66 ab	3.66 ab	3.76 a	3.69 ab
2014–2015	1.11 f	1.41 e	1.52 d	1.67 d	1.78 c	1.84 b	1.94 a	2.68 c	3.16 bc	3.13 bc	3.34 b	3.34 b	3.51 a	3.49 a
2015–2016	1.84 c	1.86 c	1.89 c	2.08 b	2.08 b	2.33 a	2.35 a	3.09 d	3.53 c	3.59 bc	3.64 bc	3.76 b	3.88 a	3.85 a
2016–2017	1.87 c	1.99 c	1.98 c	2.01 c	2.13 c	2.37 b	2.45 a	3.12 e	3.48 d	3.58 cd	3.68 c	3.84 b	3.96 a	3.85 b
2017–2018	1.58 d	1.65 c	1.74 c	1.68 c	1.98 a	1.82 b	1.97 a	2.88 e	3.11 d	3.28 c	3.45 c	3.35 c	3.67 a	3.51 b
2018–2019	1.12 e	1.45 d	1.66 c	1.68 c	1.87 b	2.05 a	1.96 ab	2.69 f	3.12 e	3.21 d	3.37 c	3.73 a	3.47 c	3.64 b
Mean	1.51d	1.68 d	1.76 c	1.85 c	1.95 b	2.13 a	2.14 a	2.09 d	3.38 c	3.46 c	3.55 b	3.55 b	3.76 a	3.68 a
Y	*							*						
N	ns							*						
Y*N	ns							ns						

Note: Different lowercase letters within a column and different capital letters within a row or column represent significant differences (P < 0.05).Means and standard errors of three replicates are presented. The means are not significantly different within a given season when followed by the same lowercase letter using LSD at P < 0.05. “**” indicates significant difference between the two optimal N rates in dry and normal year, “ns” indicates no significant difference. The same below.

### 3.3 Effects of nitrogen fertilizer on plant dry matter accumulation in dryland wheat

The dry matter accumulation at jointing stage of dryland wheat was significantly reduced by 12.9% at the N150 kg ha^−1^ compared to N210 kg ha^−1^. The increased of nitrogen fertiliser, dry matter accumulation at anthesis was non-significant variation, about 8.1–8.6 kg ha^−1^. Associated to N fertiliser rate N210 kg ha^−1^, N150 kg ha^−1^ significantly reduced dry matter accumulation at jointing stage by 12.7%, with the rise of nitrogen fertiliser level, dry matter accumulation at anthesis was non-significant variation, which was about 10.1–10.9 kg ha^−1^ (Table 1S). Nitrogen fertilizer N150 kg ha^−1^ and N180 kg ha^−1^ was additional helpful to dry matter accumulation in dryland.

The dry matter buildup of wheat at mature phase was significantly improved by 10.7% at N150 kg ha^−1^ compared to N210 kg ha^−1^ dryland wheat harvest index was rised to 5.5%, Compared to N210 kg ha^−1^, N180 kg ha^−1^ nitrogen fertilizer significantly improved dry matter buildup in dryland at maturity by 9.6%. The dryland wheat harvest index was better by 4% (Table 2S). Nitrogen fertiliser rate N150 kg ha^−1^ significantly increased dry matter buildup from jointing to flowering in dryland wheat by 14.8% compared to N fertiliser rate N210 kg ha^−1^. The dry matter buildup of anthesis to maturity of wheat in dryland was significantly improved by 34.5%. N210 kg ha^−1^ compared to N180 kg ha^−1^ significantly increased dry matter accumulation from jointing to flowering in dryland by 9%. The dry matter buildup of anthesis to maturity of wheat in dryland was significantly better by 34.3%.

### 3.4 Effects of N fertiliser rate on nitrogen accumulation of plants at various after anthesis in dryland wheat

N fertiliser rate N150 kg ha^−1^ was 19.4% lesser than that of N210 kg ha^−1^ at jointing phase. Decreased plant nitrogen accumulation at flowering stage, up to 11.7%. Significantly reduced the nitrogen accumulation at mature stage, 12.1%. N fertiliser rate N210 kg ha^−1^ compared to N180 kg ha^−1^ significantly reduced the nitrogen accumulation at jointing phase of dryland wheat by 9.8%. At flowering stage by 10.7 and at maturity stage by 6% (Table 3S). N fertiliser rate N150 kg ha^−1^, and N fertiliser rate N180 kg ha^−1^ that was, reducing N fertilizer reduced the nitrogen buildup of wheat in dryland at different growth stage.

N fertiliser rate N150 kg ha^−1^ significantly reduced leaf nitrogen accumulation by 25% related with N210 kg ha^−1^ (Table 4S), with the rise of nitrogen application rate, there were no significant variance in nitrogen accumulation of Stem + leaf sheath and cob + glume at maturity, which were 20.2–25.2 and 3.5–4.1 kg ha^−1^, respectively. N fertiliser rate N210 kg ha^−1^ compared to N180 kg ha^−1^ were increase leaf nitrogen accumulation in dryland at maturity by 3%. Significantly decreased the accumulation of cob + glume nitrogen by 24% in dryland at maturity. Increased of nitrogen application rate, N0 any significant variance in nitrogen accumulation of stem + leaf sheath at maturity, which was about 23.3–27.7 kg ha^−1^.

**Table 4:** Effects of N fertiliser rate on grain yield, and its components in dryland wheat.

Year	Grain yield (kg ha^−1^)							Spike number ( × 10^4^ ha^−1^)						
	N0	N90	N120	N150	N180	N210	N240	N0	N90	N120	N150	N180	N210	N240
2011–2012	2.29 d	3.38 c	3.90 b	4.34 a	4.26 a	4.24 a	4.23 a	391.1 e	416.7 d	447.1 c	485.5 a	476.7 b	456.3 c	457.6 c
2012–2013	3.50 e	4.84 d	5.47 c	5.45 b	5.74 a	5.48 b	5.47 b	541.3 d	566.7 c	621.2 b	630.3 b	654.0 a	626.6 b	629.7 b
2013–2014	3.54 d	4.35 c	5.23 b	5.53b	5.60 a	5.80 a	5.65 a	529.2 e	573.3 d	604.5 c	629.5 b	690.2 a	676.8 a	671.1 a
2014–2015	2.46 f	3.36 e	4.00 d	4.49 a	4.26 b	4.16 c	4.11 c	422.1 d	438.2 c	467.2 b	486.2 a	472.1 b	453.1 b	450.2 b
2015–2016	2.87 d	4.30 c	5.01 b	5.24 a	5.18 ab	5.14 ab	5.07 ab	405.3 d	485.0 c	521.6 b	551.0 a	568.1 a	522.0 b	525.1 b
2016–2017	3.18 d	4.48 c	5.34 b	5.45 b	5.55 a	5.30 b	5.31 b	437.3 d	496.8 c	501.6 c	520.8 b	573.0 a	525.1 b	529.8 b
2017–2018	2.33 d	3.16 c	4.01 b	4.63 a	4.50 a	4.39 ab	4.38 ab	381.5 d	423.2 c	481.5 b	506.0 a	498.4 a	469.0 b	470.2 b
2018–2019	2.84 d	3.85 c	4.42 b	4.85 a	4.74 a	4.65 a	4.50 ab	433.5 d	491.7 c	525.5 b	554.5 a	548.1 a	515.0 b	518.4 b
Mean	2.88 g	3.96 f	4.65 e	5.02 a	4.98 b	4.93 c	4.87 d	442.6 e	486.2 d	521.3 c	546.7 ab	559.7 a	529.7 b	531.5 b
Year	Grains per spike							1000 grains weight (g)						
	N0	N90	N120	N150	N180	N210	N240	N0	N90	N120	N150	N180	N210	N240
2011–2012	23.3 b	26.3 ab	28.3 ab	29.4 a	28.1 ab	27.1 ab	27.1 ab	35.1 a	35.6 a	35.8 a	38.1 a	40.5 a	38.4 a	38.7 a
2012–2013	30.0 b	33.2 a	32.8 a	34.4 a	35.7 a	34.4 a	33.7 a	35.5 a	38.1 a	40.3 a	41.0 a	40.1 a	40.4 a	40.8 a
2013–2014	24.5 b	29.3 a	29.9 a	29.8 a	30.8 a	30.6 a	30.8 a	39.6 c	41.2 b	42.4 b	44.4 a	43.5 a	45.8 a	41.8 b
2014–2015	30.2 a	29.8 a	30.5 a	31.1 a	30.2 a	31.8 a	30.8 a	33.5 a	34.3 a	34.8 a	36.8 a	36.7 a	35.6 a	34.7 a
2015–2016	26.5 b	29.8 ab	31.6 a	33.6 a	32.0 a	31.6 a	30.8 a	36.6 a	39.9 a	41.6 a	41.7 a	40.7 a	40.8 a	41.0 a
2016–2017	28.3 b	29.6 ab	30.2 ab	32.7 a	33.6 a	31.8 a	31.8 a	34.7 a	40.3 a	42.6 a	42.9 a	41.3 a	41.8 a	41.4 a
2017–2018	24.7 b	27.4 ab	30.7 a	31.8 a	30.8 a	31.5 a	30.4 a	33.8 b	33.6 a	35.6 a	37.4 a	38.3 a	38.3 a	37.8 a
2018–2019	25.1 b	30.6 ab	30.9 ab	32.4 a	32.1 a	31.2 a	30.6 ab	36.1 a	40.8 a	41.3 a	41.4 a	42.1 a	41.8 a	41.2 a
Mean	26.2 c	30.4 b	30.3 ab	31.5 a	31.5 a	30.8 a	30.6 ab	35.7 d	37.8 c	40.2 b	40.4 a	40.7 a	40.5 a	39.9 b
Y	**/*							*/*						
N	**/**							*/*						
Y*N	*/*							*/*						

Note: Different lowercase letters within a column and different capital letters within a row or column represent significant differences (*P* < 0.05).

After flowering the N fertiliser rate N150 kg ha^−1^ significantly increased post-anthesis nitrogen uptake by 28.7% compared to N fertiliser rate N210 kg ha^−1^. Significantly reduced the transshipment of nitrogen before flowering stage by 13%. The contribution rate of pre-anthesis transport nitrogen to grain was decreased by 5%. N fertiliser rate N210 kg ha^−1^, compared to N180 kg ha^−1^ significantly increased post-anthesis nitrogen uptake of dryland wheat by 37%. The transshipment of nitrogen before anthesis significantly reduced by 15%. The influence rate of pre-anthesis transport nitrogen to grains was significantly reduced 14.3% (Table 5S). N fertiliser rate N150 kg ha^−1^ in N180 kg ha^−1^ in year was not conducive pre-anthesis nitrogen transport in dryland wheat, but was beneficial to post-anthesis nitrogen absorption.

**Table 5:** Effects of N fertiliser rate on grain protein concentration under different nitrogen input rates of dryland wheat.

Year	Albumin (%)							Globulin (%)							Gliadin (%)						
	N0	N90	N120	N150	N180	N210	N240	N0	N90	N120	N150	N180	N210	N240	N0	N90	N120	N150	N180	N210	N240
2011–2012	1.73d	1.75d	1.82c	2.1b	2.1b	2.17 ab	2.22a	1.24b	1.26b	1.29b	1.31b	1.35b	1.42a	1.47a	3.89d	3.97c	4.06c	4.18b	4.26a	4.28a	4.32a
2012–2013	1.84d	2.26c	2.41b	2.53a	2.61a	2.59a	2.6a	1.36b	1.4b	1.51a	1.54a	1.58a	1.58a	1.61a	3.16e	3.24e	3.34d	3.58c	3.72b	3.81a	3.89a
2013–2014	1.86e	2.01d	2.15c	2.26c	2.36b	2.53a	2.62a	1.33d	1.47c	1.51b	1.52b	1.54 ab	1.59a	1.64a	3.04d	3.16c	3.26c	3.42b	3.55b	3.81a	3.85a
2014–2015	1.88d	1.93c	1.96c	2.13bc	2.2b	2.27b	2.32a	1.14d	1.21cd	1.29c	1.38b	1.41 ab	1.43a	1.46a	3.88d	3.96c	4.03c	4.16b	4.24a	4.26a	4.29a
2015–2016	1.96d	2.02c	2.25b	2.44a	2.46a	2.47a	2.48a	1.3d	1.35c	1.46c	1.51b	1.54b	1.6a	1.63a	3.23e	3.37d	3.55c	3.71b	3.82a	3.89a	3.98a
2016–2017	1.8d	2.16c	2.31b	2.47 ab	2.57a	2.58a	2.61a	1.34c	1.39c	1.48b	1.53 ab	1.55a	1.58a	1.59a	3.12e	3.27d	3.45c	3.6b	3.75b	3.84a	3.96a
2017–2018	1.83d	1.94c	2.01c	2.18b	2.22b	2.45a	2.5a	1.16d	1.2c	1.25c	1.34b	1.38 ab	1.41 ab	1.45a	3.54e	3.67d	3.78c	3.88c	3.95b	4.15b	4.26a
2018–2019	1.92c	2.06c	2.21b	2.37 ab	2.42a	2.49a	2.5a	1.15d	1.28c	1.37b	1.44 ab	1.51a	1.53a	1.48a	3.24d	3.53c	3.65b	3.7b	3.9 ab	3.98a	4.09a
Mean	1.85e	2.02d	2.14c	2.31b	2.37b	2.44a	2.48a	1.25e	1.32d	1.39c	1.44bc	1.48b	1.52a	1.54a	3.39c	3.52bc	3.64b	3.78b	3.91 ab	4.01a	4.08a
Year	Glutenin (%)							Glu/Gli							Grain protein concentration (%)						
	N0	N90	N120	N150	N180	N210	N240	N0	N90	N120	N150	N180	N210	N240	N0	N90	N120	N150	N180	N210	N240
2011–2012	3.37e	3.49e	3.62d	3.95c	4.05c	4.23b	4.35a	0.86c	0.87c	0.89c	0.94b	0.95a	0.98a	1.01a	14.7a	13.8c	13.8c	14.1b	14.2b	14.5a	14.1b
2012–2013	3.31c	3.4b	3.53b	3.79a	3.87a	3.9a	3.95a	1.04a	1.04a	1.05a	1.05a	1.03a	1.02a	1.01a	14.5a	14.1 ab	13.9 ab	13.3c	13.7b	14.2 ab	14.2 ab
2013–2014	3.33f	3.45e	3.56d	3.7c	3.87b	3.9b	4.01a	1.09a	1.09a	1.09a	1.08a	1.09a	1.02b	1.03b	14.4a	13.9c	13.4d	13.7c	13.7c	14.1b	14.1b
2014–2015	3.84c	4.06b	4.14b	4.19b	4.24a	4.3a	4.31a	0.98b	1.02a	1.02a	1.01a	1.01a	1.01a	1.01a	14.8a	14.6a	13.6b	13.9b	14.2 ab	14.5 ab	14.4 ab
2015–2016	3.36f	3.44e	3.59d	3.75c	3.92b	4.03b	4.1a	1.03a	1.02a	1.01a	1.01a	1.02a	1.03a	1.03a	14.7a	13.9b	13.6c	13.6c	14.1 ab	14.3 ab	14.4 ab
2016–2017	3.39f	3.45e	3.59d	3.85c	3.93b	4.01b	4.07a	1.08a	1.05b	1.04c	1.06b	1.04c	1.04c	1.02d	15.3a	14.3b	13.9c	14.1c	14.1c	14.4b	14.5b
2017–2018	3.27g	3.4f	3.52e	3.85d	3.95c	4.12b	4.26a	0.92b	0.92b	0.92b	0.99a	1.01a	0.99a	1.01a	14.6a	13.9b	13.3c	13.2c	13.2c	13.5b	13.8b
2018–2019	3.18f	3.36e	3.46d	3.53c	3.61c	3.91b	4.05a	0.98a	0.95b	0.94c	0.95b	0.92c	0.98a	0.98a	14.5a	13.9b	13.6b	13.2c	13.1c	13.7b	13.7b
Mean	3.38f	3.51e	3.62d	3.82c	3.93c	4.05b	4.14a	1.01a	1.01a	0.99a	1.01a	1.01a	1.01a	1.01a	14.6a	14.1b	13.6c	13.6c	13.7c	14.2b	14.2b
ANOVA																					
Y	*/*							*/*							*/*						
N	*/**							**/*													
Y*N	*/*							*/ns							*/ns						

Note: Different lowercase letters within a column and different capital letters within a row or column represent significant differences (*P* < 0.05).Means and standard errors of three replicates are presented.

### 3.5 Effects of nitrogen fertilizer on yield component, and protein content in dryland wheat

The highest spike number was 486.6 × 10^4^ ha^−1^ respectively. Moreover, significant variance between the nitrogen application rate of N150 kg ha^−1^ in 2009–2010, 2012–2013, 2015–2016 and 2016–2017 and the nitrogen application rate of N180 kg ha^−1^ in addition (Table 4). The highest spike number was 655.1 × 10^4^ ha^−1^, 689.1 × 10^4^ ha^−1^, 569 × 10^4^ ha^−1^, 574.1 × 10^4^ ha^−1^, respectively. There was a significant variance b/w N fertiliser rate N180 kg ha^−1^ and other N fertiliser rate in 2010–2011 and 2014–2015, and between 180 kg ha^−1^ and N0, N90, N120 and N150 kg ha^−1^ in 2011–2012. In 2013–2014, the N applications level of N180 kg ha^−1^ was significantly different from that N fertiliser rate N0, N90, N120, N210, and N240 kg ha^−1^.

N application grain number per spike was the highest at 150 kg ha^−1^, which was significantly variance from N fertiliser rate N0 kg ha^−1^ (Table 4). In 2010–2011, 2011–2012 and 2014–2015, the N fertiliser rate N180 kg ha^−1^ was the highest, which was significantly changed from N0 kg ha^−1^. From 2013 to 2014, the highest N application rate was N150 kg ha^−1^ per grain per spike, which was significantly different from N fertiliser rate N0 kg ha^−1^. The thousand grain weight was the highest with nitrogen application rate of N180 kg ha^−1^, and the variance between N fertiliser rate N180 kg ha^−1^, and N0 kg ha^−1^ was significant just in 2015–2016. The thousand grain weight was the highest with N fertiliser rate N150 kg ha^−1^ in 2010–2011, 2013–2014 and 2014–2015, but there was no significant difference founded with other nitrogen application rates. From 2011 to 2012, the thousand grain weight of N fertiliser rate N210 kg ha^−1^ was the highest, reaching 46.9 g, which was significantly diverse from N0, N90, N120, and N240 kg ha^−1^.

The spike number was increased by 7.6% at N fertiliser rate N150 kg ha^−1^ compared to N210 kg ha^−1^, with the increase of nitrogen application rate, grain number per spike, and thousand grain weight did not change significantly. N fertiliser rate N210 kg ha^−1^, the spike number was increased by 5.3% at N180 kg ha^−1^. N application rate, grain number per spike, and thousand grain weight was non-significant modification. The contents of albumin, globulin and glutenin in dryland wheat grains were no significant differences, which were 2.19–2.34%, 1.37–1.44%, and 3.98–4.17%, respectively (Table 5). Associated to N fertiliser rate N210 kg ha^−1^, N150 kg ha^−1^ significantly reduced grain glutenin content in dryland wheat. With the rise of nitrogen application level, the contents of albumin, globulin and glutenin in dryland wheat grains was non-significant difference, which were albumin (2.42–2.54%), globulin (1.52–1.59%) and glutenin (3.77–3.96%), respectively. N fertiliser rate N210 kg ha^−1^ compared to N180 kg ha^−1^ significantly reduced grain glutenin content in dryland wheat.

### 3.6 Nitrogen use efficiency and nitrogen partial productivity

The NUE and NPP of dryland wheat were significantly increased by 36% and 32%, respectively. N180 kg ha^−1^ compared to N210 kg ha^−1^, N application rate of significantly increased NUE and N partial productivity of dryland wheat by 17% and 15%, respectively (Table 6). In conclusion, N150 kg ha^−1^ significantly improved the NUE, and nitrogen partial productivity of dryland wheat in all years.

**Table 6:** Effects of nitrogen rate on nitrogen use efficiency index in dryland winter wheat.

Year	Nitrogen use efficiency (NUE) (kg kg^−1^)							Nitrogen partial factor productivity (PFP) (kg kg^−1^)						
	N0	N90	N120	N150	N180	N210	N240	N0	N90	N120	N150	N180	N210	N240
2011–2012	–	12.1b	13.4a	13.6a	10.9c	9.2d	8.1e	–	37.6a	32.5b	29c	23.7d	20.2e	17.6f
2012–2013	–	14.6b	16.4a	13c	12.4d	9.4e	8.2f	–	53.6a	45.6b	36.4c	31.9d	26.1e	22.8f
2013–2014	–	9e	14a	13b	11.3c	10.7d	8.7f	–	48.5a	43.6b	36.8c	31.1d	27.6e	23.5f
2014–2015	–	10c	12.8b	13.5a	10.0c	8.1d	6.8e	–	37.4a	33.4b	30c	23.7d	19.8e	17.1f
2015–2016	–	15.8b	17.6a	15.8b	12.8c	10.8d	9.2e	–	47.8a	41.6b	35c	28.8d	24.5e	21.1f
2016–2017	–	14.4b	18a	15.1b	13.1c	10.1d	8.8e	–	49.8a	44.5b	36.4c	30.8d	25.2e	22.1f
2017–2018	–	9.2c	13.8b	15.3a	12bc	9.8c	8.5d	–	35.2a	33.3b	30.9c	25d	20.9e	18.2f
2018–2019	–	11.2c	13.1b	13.4a	10.5d	8.6e	6.9f	–	42.8a	36.9b	32.4c	26.3d	22.1e	18.7f
Mean	–	12b	14.7a	14.2a	11.6b	9.7c	8.2d	–	44.1a	38.8b	33.5c	27.72d	23.5e	20.3f
Y		*	*	*	*	*	*		*	*	*	*	*	*
N		**	**	**	**	**	**		*	*	*	*	*	*
Y*N		*	*	*	*	*	*		*	*	*	*	*	*

Note: Different lowercase letters within a column and different capital letters within a row or column represent significant differences (*P* < 0.05).Means and standard errors of three replicates are presented. The means are not significantly different within a given season when followed by the same lowercase letter using LSD at *P* < 0.05. “**” indicates significant difference between the two optimal N rates in dry and normal year, “ns” indicates no significant difference. The same below.

Note: NUE and PFP represent Nitrogen use efficiency and Nitrogen partial factor productivity.

### 3.7 Effects of N fertiliser rate on wheat yield and its components in dryland wheat

**Fig. 5:**
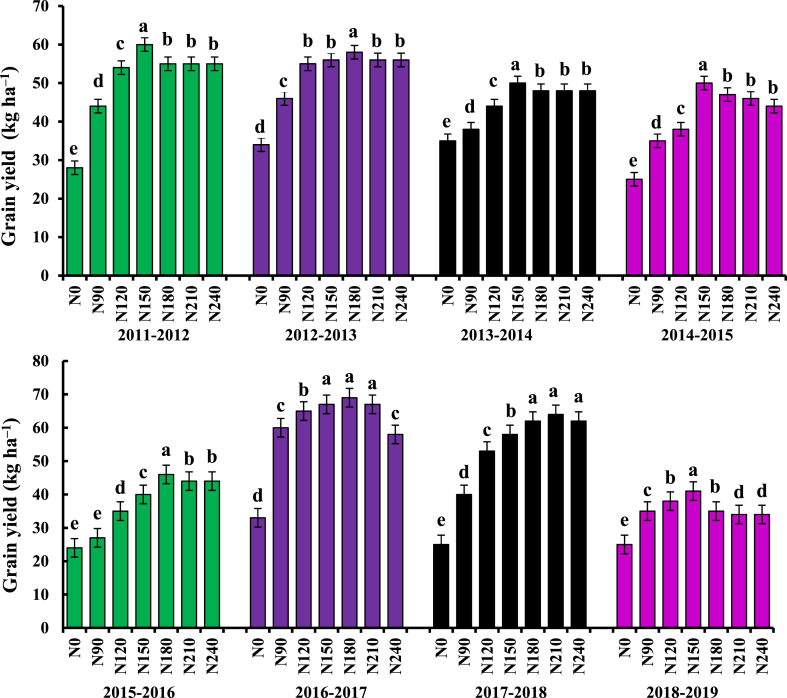
Effects of nitrogen fertilizer on grain yield in dryland wheat. (Upper-case letters and lower-case letters indicate comparisons between N rates between. letter means significant at 0.05 probability levels. Bars represent the standard error).

The highest yield of wheat were 4.35 kg ha^−1^, 4.50 kg ha^−1^, 4.64 kg ha^−1^ and 4.86 kg ha^−1^ at the N fertiliser rate N150 kg ha^−1^, respectively. The N fertiliser rate N150 was significantly different from that of N0, N90 and N fertiliser rate N120 kg ha^−1^, and N150 kg ha^−1^ was significantly different from other nitrogen treatments (Fig. 5). The yield of upland wheat in 2010–2011, and 2014–2015 was significantly higher at N fertiliser rate N180 kg ha^−1^, reaching 5.75 kg ha^−1^ and 5.56 kg ha^−1^, respectively. From 2011 to 2012, the highest yield was 5.81 kg ha^−1^ at the N fertiliser rate N210, which was significantly different N fertiliser rate N0, N90, N120 and N150 kg ha^−1^. From 2018 to 2019, the highest yield was 5.25 kg ha^−1^ with N fertiliser rate of N150 kg ha^−1^, which was significantly different from N0, N90 and N120.

## 4 Discussion

The synergistic effects of fertilizer, and soil moisture determines wheat yield. Insufficient soil moisture reduces the positive effects of soil nitrogen on wheat production, while heavy precipitation or excessive irrigation amount will lead to leaching loss of soil nitrogen, which will also have adverse effects on yield [16]. Soil Water deficit at tillering tended to increase grain harvest index but decreased biomass, it would have a serious effects on spike number. If it appeared in the booting to flowering stage, it significantly affected the grain number per spike of wheat [17]. Higher soil nitrogen nutrient level will accelerate plant growth, thus depleting soil water reserve. Although wheat can produce more grains, its grains may not be full because of soil water accumulation [18]. The order to get advanced grain yield, and crop water production proficiency, nitrogen fertilizer input must adjusted according to precipitation. Therefore, scientific fertilization must consider the accumulation, and utilization of water. N fertiliser rate N210 kg ha^−1^ compared to N150 kg ha^−1^ significantly increased the water use efficiency of crops by 6.1%. It indicated that one of the reasons for high yield in different precipitation year types was higher water use efficiency [19]. The precipitation fallow use efficiency (PFUR) was equal to the fallow soil water storage ratio (SWSR), indicating that the fallow precipitation stored in the soil could only meet a part of the water consumption demand of subsequent crops. The soil water accumulated during fallow period could meet the water consumption before jointing stage in dryland wheat. In the whole year, the soil water accumulated during fallow period can meet the water consumption before anthesis of dryland wheat. This is similar to the conclusion proposed by Ref. [20]. That precipitation in the leisure period can affect the jointing stage [21]. Research on the spatio-temporal dynamics of water in dryland wheat fields in Shanxi N fertiliser rate N210 kg ha^−1^ significantly improved soil water consumption in 80/240 cm soil layer since jointing to flowering by 36.6% related to that of N210 kg ha^−1^. There was a significant positive parallel between soil water intake and spike number between jointing to flowering 80, 240 cm (a = 1.7, R2 = 0.45, *P* < 0.001). N210 compared to soil water consumption of N180 kg ha^−1^ significantly increased by 22.7% in the 80–240 cm soil layer, and there was a significant positive correlation between the soil water consumption of N180 t ha^−1^ and the spike number (a = 2.9, R2 = 0.47, *P* < 0.001). The N240 kg ha^−1^ significantly increased the water consumption of jointing to flowering soil in dryland, promoted tiller spike creation, improved the actual spike number, and then increased the yield.

The process of crop growth progress, and yield creation is the process of material transformation between crops, and the environment, as well as the process of material accumulation and transformation between crop organs. Smooth material accumulation, and carriage in the key development period is the essence crops to achieve high yield [22,23]. The influence of precipitation at various growth stages on wheat yield in dryland was priority order of sowing stage to jointing stage other growth stages, and the main limiting factor of wheat yield in dryland was precipitation at sowing emergence, and jointing stage [24]. The key growth period of tiller differentiation in dryland wheat is before after jointing stage, which also has an important influence on spike number at mature stage [5,25]. Believed that ineffective tiller would reduce the optimal effective population number of wheat, and excessive ineffective tiller would also have a negative influence on the amount of ear, and grain weight of wheat. As a result spike number of studies have focused on delaying wheat jointing stage tillering polarization process, the method of the size of the population will affects the growth, and development of wheat individuals nutrient competition environment and resources, thus affecting wheat tiller earing rate of late, has a large influence on yield, and its components [26]. Affects of soil nitrogen, and water conditions in wheat fields at jointing stage. The N percentage of wheat was significantly increased by 20.3% with N application rate of 150 kg ha^−1^ compared to 210 kg ha^−1^. Compared to 210 kg ha^−1^, the tiller of dryland wheat was significantly increased by 7% at N180 kg ha^−1^. Nitrogen application rate of N150 kg ha^−1^ significantly improved dry matter buildup from jointing to flowering in dryland wheat by 14.8% compared with 210 kg ha^−1^ [20]. Comparing to 210 kg ha^−1^, 180 kg ha^−1^ significantly increased the dry matter buildup of jointing to flowering wheat in dryland by 9%. Connection analysis showed that dry matter accumulation from jointing to flowering was significantly positively correlated with tiller percentage in dryland [25]. Suitable nitrogen application rate can significantly increase dry matter accumulation from jointing to flowering stage, increase tiller rate, promote the formation of effective tiller, and increase yield. The relationship between sources is the material basis for yield formation. The results presented that grain profit decreased with the rise of dry matter buildup (A = 11.1, R2 = 0.50, *P* < 0.05; A = 17.4, R2 = 0.65, *P* < 0.01) [27]. In Shanxi secretary-general of long-term experimental study show that the output of rainfed area precipitation effect than fertilizer, especially the leisure precipitation, regardless of the leisure period precipitation in fertilizer inputs [28]. Study the significant non-linear positive correlation between precipitation in fallow period and yield, and proposed a yield prediction model for precipitation in fallow period to simulate the yield performance of precipitation in different fallow periods [29]. The idea is to explore the linear or nonlinear association b/w rainfall and yield in fallow period, and to calculate the quantity of fertilizer obligatory for yield calculation. In this study, the distribution features of rain in fallow period in dryland wheat were examined, and the annual types were divided by rainfall in fallow period, and the nitrogen application amount for changed annual types of precipitation in fallow period was explored by field experiments with altered nitrogen gradients. The results showed N application rate of N150 kg ha^−1^ was significantly improved by 5.0% compared to N210 was decreased by 28.6%. N210 kg ha^−1^, compared to N180 kg ha^−1^ significantly increased wheat yield in dryland by 5.5%, while 30 kg ha^−1^ nitrogen fertilizer was reduced by 14.3%. The spike number increased by 7.6% N150 kg ha^−1^ compared to N210 kg ha^−1^, but there were non-significant differences in grain number per spike and 1000-grain weight. Compared to N210 kg ha^−1^ with N180 kg ha^−1^ significantly increased spike number by 5.3%, but there was no significant difference in grain number per spike and 1000-grain weight [30]. The eight years experiment in Changwu, Shanxi presented that the soil moisture before hand sowing in dryland wheat was less than 200 mm, 200–250 mm and >300 mm, and the reasonable range of nitrogen application were 83–105 kg ha^−1^, 98–120 kg ha^−1^ and 105–135 kg ha^−1^. The results were lower from present study, and the differences in climatic conditions and soil fertility in different test areas may lead to differences between studies [31]. The reasonable nitrogen application amounts of N180 kg ha^−1^ were similar to the effects of this study when the annual precipitation was normal (500–600 mm) and humid (600 mm). In the dry farming wheat region of the Loess Plateau, the annual types are divided according to the precipitation during the fallow period, which can be adjusted based on the local precipitation data in each region to be applicable to the local production reality [32]. The results presented that N150 kg ha^−1^ significantly reduced grain protein contented of dryland wheat by 0.5% compared to N210 kg ha^−1^ compared to N210 kg ha^−1^, N180 kg ha^−1^ significantly reduced grain protein of dryland wheat by 0.6%. The results indicated that suitable nitrogen application based on fallow precipitation reduced grain protein content of dryland wheat, and further screening and exploring the potential of nitrogen utilization of dryland wheat varieties would be one of the effective directions.

## 5 Conclusions

This study carried out a study on the effects of nitrogen fertilizer on soil water nitrogen utilization, and yield formation of dryland wheat in different rainfall years. In order to provide theoretical basis and technical support for high-yield and high-efficiency production in dryland wheat area of loess Plateau. According to the annual classification technique of rainfall in fallow period, were classified as perennial years. Related to N210 kg ha^−1^, 150 kg ha^−1^ reduced N and increased yield. All year round, related to N210 kg ha^−1^, N180 kg ha^−1^ reduced N and increased yield. The dry substance buildup from jointing to flowering was improved respectively, while grain protein content was significantly decreased. Compared to N210 kg ha^−1^, the annual nitrogen submission rate of N180 kg ha^−1^ significantly better soil water intake in 80–240 cm soil layer from jointing to flowering. N210 kg N ha^−1^ was mainly responsible for a reduction in 1000-grain weight and grain number per spike. Therefore, in high-yield years, fallow cultivation can help adjust the relationship among the components, promote a reasonable distribution, and improve yield.

## Author contribution statement

All authors listed have significantly contributed to the development and the writing of this article.

## Data availability statement

No data was used for the research described in the article.

## Funding statement

This study was supported by the National Key R&D Program of China (2021YFD1900700) for financial support of this study.

## Declaration of competing interest

The authors declare that they have no known competing financial interests or personal relationships that could have appeared to influence the work reported in this paper.

## References

[1] Fan Y.L., Liu J.M., Zhao J.T., Li Q.Q. (2019). Effects of delayed irrigation during the jointing stage on the photosynthetic characteristics and yield of winter wheat under different planting patterns. Agric. Water Manag..

[2] Li W.Q., Han M.M., Pang D.W., Jin C., Wang Y.Y., Dong H.H., Chang Y.L. (2022). Characteristics of lodging resistance of high-yield winter wheat as affected by nitrogen rate and irrigation managements. J. Integr. Agric..

[3] Manschadi A.M., Soltani A. (2021). Variation in traits contributing to improved use of nitrogen in wheat: implications for genotype by environment interaction. Field Crops Res..

[4] Cao H.B., Wang Z.H., He G., Dai J., Huang M., Wang S. (2017). Tailoring NPK fertilizer rates to precipitation for dryland winter wheat in the Loess Plateau. Field Crops Res..

[5] McMaster G.S., Wilhelm W.W. (2004). Phenological responses of wheat and barley to water and temperature: improving simulation models. J. Agric. Sci..

[6] Noor Hafeez, Sun Min, Lei Bin, Gao Zhi-Qiang (2022). Disadvantages of sowing methods on soil water content root distribution and yield of wheat (*Triticum aestivum* L.) in the Loess Plateau of South Shanxi, China. Water Supply.

[7] Shangguan Z.P., Shao M.A., Lei T.W., Fan T.L. (2002). Runoff water management technologies for dryland agriculture on the Loess. Int. J. Sustain. Dev. World Ecol..

[8] Li S.X., Wang Z.H., Malhi S.S., Li S.Q., Gao Y.J. (2009). Nutrient and water management effects on crop production, and nutrient and water use efficiency in dryland areas of China. Adv. Agron..

[9] Guo S., Zhu H., Dang T., Wu J., Liu W., Hao (2012). Winter wheat grain yield associated with precipitation distribution under long-term nitrogen fertilization in the semiarid Loess Plateau in China. Geoderma.

[10] Carolina, Rivera-Amado, Eliseo, grellos T.-N., Gemma Molero, Matthew P., Reynolds Roger (2019). Optimizing dry-matter partitioning for increased spike growth, grain number and harvest index in spring wheat - ScienceDirect. Field Crops Res..

[11] Noor Hafeez, Sun M., Lin W., Gao Z. (2022). Effect of different sowing methods on water use efficiency and grain yield of wheat in the Loess Plateau, China. Water.

[12] Liu X., Wang W.X., Wang D. (2020). The effects of intraspecific competition and light transmission within the canopy on wheat yield in a wide-precision planting pattern. J. Integr. Agric..

[13] Liu X.H., Ren Y.J., Gao C., Yan Z.X., Li Q.Q. (2017). Compensation effect of winter wheat grain yield reduction under straw mulching in wide-precision planting in the North China Plain. Sci. Rep..

[14] Laza M.R., Peng S., Akita S., Saka H. (2003). Contribution of biomass partitioning and translocation to grain yield under sub-optimum growing conditions in irrigated rice. Plant Prod. Sci..

[15] Cox M.C., Qualset C.O., Rains D.W. (1986). Genetic variation for nitrogen assimilation and translocation in wheat. Nitrogen translocation in relation to grain yield and protein. Crop Sci..

[16] Basso B., Fiorentino C., Cammarano, Dardanelli J. (2012). Analysis of rainfall distribution on spatial and temporal patterns of wheat yieldin Mediterranean environment. Eur. J. Agron..

[17] Hochman Z. (1982). Effect of water stress with phasic development on yield of wheat grown in a semi-arid environment. Field Crops Res..

[18] Fan M., Shen J., Yuan L., Jiang R., Chen X., Davies W.J., Zhang F. (2012). Improving crop productivity and resource use efficiency to ensure food security and environmental quality in China. J. Exp. Bot..

[19] Chen Z.M., Wang H., Liu X., Lu D., Zhou J. (2016). The fates of N-labeled fertilizer in a wheat–soil system as influenced by fertilization practice in a loamy soil. Sci. Rep..

[20] Noor H., Wang Q., Sun M., Fida N., Gao Z.Q. (2021). Effects of sowing methods and nitrogen rates on photosynthetic characteristics, yield and quality of winter wheat. Photosynthetica.

[21] Sun M., Ren A.X., Gao Z.Q., Wang P.R., Mo F., Xue L.Z. (2018). Long-term evaluation of tillage methods in fallow season for soil water storage, wheat yield and water use efficiency in semiarid southeast of the Loess Plateau. Field Crops Res..

[22] Ren A., Sun M., Xue L., Deng Y., Wang P., Lei M., Xue J. (2019). Spatio-temporal dynamics in soil water storage reveals effects of nitrogen inputs on soil water consumption at different growth stages of winter wheat. Agric. Water Manag..

[23] He G., Wang Z., Li F., Dai J., Li Q., Xue C. (2016). Soil water storage and winter wheat productivity affected by soil surface management and precipitation in dryland of the Loess Plateau, China. Agric. Water Manag..

[24] Jia J.Y., Zhao J.F., Wan X., Han L.Y., Wang X.W., Liang Y. (2017). Effects of soil water storage efficiency on winter wheat water use efficiency in different precipitation areas during the fallow period in the Loess Plateau, western China. Acta Ecol. Sin..

[25] Foulkes M.J., Hawkesford M.J., Holdsworth M.J., Kerr S., Kightley S., Shewry P.R. (2009). Identifying traits to improve the nitrogen economy of wheat: recent advances and future prospects. Field Crops Res..

[26] Wang X., Tong Y., Liu F., Zhao Z., Pang Y. (2014). Spatial and temporal variations of crop fertilization and soil fertility in the loess plateau in China from the 1970s to the 2000. PLoS One.

[27] Huang M., Dang T., Gallichand J., Goulet M. (2003). Effect of increased fertilizer applications to wheat crop on soil-water depletion in the Loess Plateau, China. Agric. Water Manag..

[28] Peng S.B., Ismail A. (2004).

[29] Xue L., Khan S., Sun M., Anwar S., Ren A., Gao Z., Lin W., Xue J. (2019). Effects of tillage practices on water consumption and grain yield of dryland winter wheat under different precipitation distribution in the loess plateau of China. Soil Tillage Res..

[30] Wang J., Liu W., Dang T. (2011). Responses of soil water balance and precipitation storage efficiency to increased fertilizer application in winter wheat. Plant Soil.

[31] Yoshida S. (1972). Physiological aspects of grain yield. Annu. Rev. Plant Physiol..

[32] Zhao D.D., Shen J.Y., Li Q.Q. (2013). Effects of irrigation and wide-precision planting on water use, radiation interception, and grain yield of winter wheat in the North China Plain. Agric. Water Manag..

