# Supplementary material for: Nitrogen fertilization and precipitation affected Wheat (Triticum aestivum L.) in dryland the Loess Plateau of South Shanxi, China
Source: Heliyon. 2023 Jul 13;9(7):e18177. doi: 10.1016/j.heliyon.2023.e18177 (PMC10375798; doi:10.1016/j.heliyon.2023.e18177)
Supplement: Multimedia component 1 [file mmc1.docx]

| **Table 1S**. Effects of N fertiliser rate on dry matter accumulation of differrent grow stages in dryland winter wheat. | | | | | | | | | | | | | | | | | | | | | |
| --- | --- | --- | --- | --- | --- | --- | --- | --- | --- | --- | --- | --- | --- | --- | --- | --- | --- | --- | --- | --- | --- |
|  |  |  |  |  |  |  |  |  |  |  |  |  |  |  |  |  |  |  |  |  |  |
| Year | **Jointing stage** | | | | | | | **Anthesis stage** | | | | | | | **Maturity stage** | | | | | | |
|  | N0 | N90 | N120 | N150 | N180 | N210 | N240 | N0 | N90 | N120 | N150 | N180 | N210 | N240 | N0 | N90 | N120 | N150 | N180 | N210 | N240 |
| 2011-2012 | 2.59 c | 2.74bc | 2.84b | 2.92b | 3.21a | 3.25a | 3.2a | 5.94e | 6.61d | 7.19c | 7.31b | 7.91a | 7.44b | 5.79e | 6.79e | 8.11d | 9.37b | 10.91a | 10.53b | 9.77bc | 8.82c |
| 2012-2013 | 2.50 d | 2.62d | 3.09c | 3.32b | 3.41b | 3.67a | 3.58a | 7.47d | 9.03c | 10.29b | 10.72b | 12.01a | 10.58b | 10.77b | 9.11d | 12.26c | 13.66b | 13.77b | 15.54a | 13.46b | 13.69b |
| 2013-2014 | 3.07 d | 3.27c | 3.4c | 3.5bc | 3.78b | 4.21a | 4.36a | 8.26d | 9.79c | 10.62b | 11.32ab | 11.63a | 11.9a | 11.98a | 10.44d | 11.54c | 12.34b | 12.96ab | 13.18ab | 13.67a | 13.25ab |
| 2014-2015 | 2.48 c | 2.68bc | 2.71b | 2.87b | 2.82b | 2.92a | 2.43c | 5.82d | 6.48c | 7.05b | 7.76a | 7.29ab | 7.16b | 5.68d | 7.02e | 8.58d | 9.54c | 10.44a | 10.01b | 9.24c | 8.57d |
| 2015-2016 | 2.84 c | 2.85c | 2.93c | 2.78c | 3.39b | 3.55a | 3.47a | 7.46c | 8.43b | 8.64b | 8.22b | 9.57ab | 9.46ab | 10.18a | 8.52e | 11.08d | 12.14c | 11.56cd | 13.15a | 12.52b | 13.31a |
| 2016-2017 | 2.25 f | 2.45e | 2.7d | 2.99c | 3.09c | 3.33b | 3.6a | 7.06e | 8.75d | 9.26c | 10.01b | 10.53a | 9.25c | 9.59c | 8.72e | 11.35d | 12.79c | 14.66b | 16.52a | 14.88b | 15.12ab |
| 2017-2018 | 2.73 e | 2.88d | 2.99c | 3.17b | 3.47a | 3.54a | 3.63a | 5.86d | 6.96c | 7.13c | 7.7a | 7.47b | 6.95c | 5.85d | 7.01e | 8.75d | 10.18b | 11.29a | 11.06b | 10.21b | 9.08c |
| 2018-2019 | 3.36 c | 3.54b | 3.67b | 3.53b | 3.59b | 3.91a | 3.93a | 6.73d | 8.55b | 8.75b | 9.18a | 9.46a | 8.54b | 7.18c | 8.29e | 10.38c | 11.13c | 12.74a | 12.56a | 12.34ab | 10.21d |
| Mean | 2.72 f | 2.87e | 3.04d | 3.13c | 3.34b | 3.54a | 3.54a | 6.07c | 7.19b | 7.67b | 8.04ab | 8.45a | 7.94ab | 7.47b | 7.32e | 9.13d | 10.14c | 10.81b | 11.41a | 10.7b | 10.25c |
| ANOVA | | | | | | | | | | | | | | | | | | | | | |
| Y | * | | | | | | | * | | | | | | | * | | | | | | |
| N | ** | | | | | | | ** | | | | | | | * | | | | | | |
| Y*N | * | | | | | | | * | | | | | | | * | | | | | | |
| Note: Different lowercase letters within a column and different capital letters within a row or column represent significant differences (*P* < 0.05). | | | | | | | | | | | | | | | | | | | | | |
|  |  |  |  |  |  |  |  |  |  |  |  |  |  |  |  |  |  |  |  |  |  |

| **Table 2S.** Effcet of N fertiliser rate on translocation of Pre-/Post anthesis accumulated dry matter to grain in dryland winter wheat. | | | | | | | | | | | | | | | | | | | | | |
| --- | --- | --- | --- | --- | --- | --- | --- | --- | --- | --- | --- | --- | --- | --- | --- | --- | --- | --- | --- | --- | --- |
|  |  |  |  |  |  |  |  |  |  |  |  |  |  |  |  |  |  |  |  |  |  |
| Year | Translocation of Pre-anthesis accumulated dry matter to grain (*W*) | | | | | | | Translocation of Post-anthesis accumulated dry matter to grain (*T*) | | | | | | | Contribution of dry matter transport before and after anthesis to grain *W% / T%* | | | | | | |
|  | N0 | N90 | N120 | N150 | N180 | N210 | N240 | N0 | N90 | N120 | N150 | N180 | N210 | N240 | N0 | N90 | N120 | N150 | N180 | N210 | N240 |
| 2011-2012 | 1.45e | 1.89b | 1.73c | 1.55d | 1.65d | 1.92b | 2.21a | 0.84g | 1.49f | 2.18d | 2.79a | 2.61b | 2.32c | 2.02e | 63/36 | 35/65 | 44/56 | 35/65 | 38/62 | 45/55 | 52/48 |
| 2012-2013 | 1.88e | 1.59f | 2.1d | 2.61b | 2.22c | 2.71a | 2.66b | 1.63e | 3.23c | 3.37bc | 3.43b | 3.52a | 2.87d | 2.91d | 53/47 | 32/67 | 38/62 | 43/57 | 38/62 | 48/52 | 47/53 |
| 2013-2014 | 1.37f | 1.62e | 2.51b | 2.87a | 2.05d | 2.03d | 2.38c | 2.18d | 2.74a | 2.72a | 2.64b | 2.75a | 2.7a | 2.27c | 38/62 | 37/63 | 47/53 | 52/48 | 42/58 | 42/58 | 51/49 |
| 2014-2015 | 1.27d | 1.27d | 1.51b | 1.52b | 1.55b | 2.09a | 1.42c | 1.19f | 2.09e | 2.49d | 2.68b | 2.57c | 2.07e | 2.89a | 51/49 | 37/63 | 37/63 | 36/64 | 37/63 | 50/50 | 32/68 |
| 2015-2016 | 1.82b | 1.65c | 1.49d | 1.9b | 1.61c | 2.08a | 1.87b | 1.05e | 2.65a | 3.5b | 3.34c | 3.57ab | 3.05d | 3.12cd | 63/36 | 38/62 | 29/71 | 36/64 | 31/69 | 40/60 | 37/63 |
| 2016-2017 | 1.33b | 1.68a | 1.62b | 1.68a | 1.6b | 1.71a | 1.69a | 1.66e | 2.59d | 3.52c | 3.65b | 3.78a | 3.62b | 3.53bc | 44/56 | 39/61 | 31/69 | 31/69 | 29/71 | 32/68 | 32/68 |
| 2017-2018 | 1.19d | 1.38c | 1.34c | 1.55b | 1.75a | 1.81a | 1.57b | 1.14e | 1.78d | 3.05c | 3.59a | 3.59a | 3.25b | 3.23b | 51/49 | 40/60 | 30/70 | 30/70 | 32/68 | 35/65 | 32/68 |
| 2018-2019 | 1.29e | 2.03a | 2.05a | 1.63d | 1.68d | 1.86b | 1.78c | 1.55g | 1.83f | 2.37e | 3.32a | 3.1c | 3.27b | 3.02d | 45/55 | 52/48 | 46/54 | 32/68 | 35/65 | 36/64 | 37/63 |
| Mean | 1.45f | 1.76e | 1.91d | 2.03c | 2.01c | 2.27a | 2.19b | 1.4d | 2.3c | 2.9b | 3.18a | 3.18a | 2.9b | 2.87b | 50/50 | 38/62 | 40/60 | 39/61 | 39/61 | 38/62 | 40/60 |
| ANOVA | | | | | | | | | | | | | | | | | | | | | |
| Y | * | | | | | | | * | | | | | | | */** | | | | | | |
| N | ** | | | | | | | ** | | | | | | | ns/ns | | | | | | |
| Y*N | ns | | | | | | | Ns | | | | | | | ns/ns | | | | | | |
| Note: （*W*）: Translocation of Pre-anthesis accumulated dry matter to grain；（*T*）: Translocation of Post-anthesis accumulated dry matter to grain；（*W%/ T%*）Rate of the translocation of Pre-anthesis accumulated dry matter to grain and Rate of translocation of Post-anthesis accumulated dry matter to grain. Different lowercase letters within a column and different capital letters within a row or column represent significant differences (*P* < 0.05). | | | | | | | | | | | | | | | | | | | | | |
|  |  |  |  |  |  |  |  |  |  |  |  |  |  |  |  |  |  |  |  |  |  |

| **Table 3S.** Effcets of N fertiliser rate on total nitrogen accumulation of different growth stages in dryland Winter Wheat. | | | | | | | | | | | | | | | | | | | | | | | |
| --- | --- | --- | --- | --- | --- | --- | --- | --- | --- | --- | --- | --- | --- | --- | --- | --- | --- | --- | --- | --- | --- | --- | --- |
|  |  |  |  |  |  |  |  |  |  |  |  |  |  |  |  |  |  |  |  |  |  |  |  |
| Year | Jointing stage | | | | | | | Anthesis stage | | | | | | | Maturity stage | | | | | | | | |
|  | N0 | N90 | N120 | N150 | N180 | N210 | N240 | N0 | N90 | N120 | N150 | N180 | N210 | N240 | N0 | | N90 | | N120 | N150 | N180 | N210 | N240 |
| 2011-2012 | 36.7f | 38.9e | 41.3d | 45.8c | 55.4b | 59.4a | 58.2a | 61.9f | 68.4e | 79.6d | 86.2c | 95.8a | 94a | 90.3b | 83.6f | | 111.3e | | 126.2d | 141.9b | 135.1c | 146.2a | 141.6b |
| 2012-2013 | 37.9f | 41.5e | 50.2d | 53.5c | 64.5b | 69a | 70.7a | 84.4g | 99.5f | 120e | 131.8d | 149.8c | 168.9a | 163.3b | 118.1f | | 158.3e | | 175.5c | 170.1d | 190.4b | 198.8a | 198.4a |
| 2013-2014 | 44.6e | 49.2d | 48.8d | 54.4c | 54.1c | 61.8b | 64.4a | 88.9f | 107.4e | 114.4d | 130.1c | 136.8b | 156.8a | 152.1a | 122.1f | | 146.1e | | 164.3d | 174.8c | 181.9b | 186.1a | 182.3b |
| 2014-2015 | 31.4c | 32.7bc | 34.1b | 33.3b | 35.3b | 38.9a | 38.6a | 74.8d | 81c | 86.2c | 99.9b | 100.2b | 114.2a | 112.1a | 94.1e | | 119.3d | | 129.4c | 146.9ab | 144.9b | 148.8a | 141.3b |
| 2015-2016 | 43.9e | 44.4e | 45.2e | 47.6d | 53.3c | 61.1b | 63.7a | 86.5d | 96.7c | 100.4c | 101.3c | 125.1b | 141.3a | 137.3a | 102.5e | | 142.1d | | 157.8c | 165.9b | 174.4ab | 176.4a | 178.2a |
| 2016-2017 | 36f | 40.2e | 45.1d | 54.0c | 58.7b | 63.7a | 64.9a | 85.1f | 106.1e | 115.8d | 128.3c | 142.8b | 154.2a | 155.8a | 113.9e | | 150.8d | | 171.8c | 182.7b | 193.2a | 195.2a | 191.4a |
| 2017-2018 | 36.1f | 38.6e | 42.9d | 48.5c | 49c | 55.9a | 51.8b | 60.2e | 69.5d | 77.4c | 86b | 88.4b | 99.4a | 91.2b | 83.7e | | 106.3d | | 126.2c | 143.6a | 132.7b | 142.9a | 142.9a |
| 2018-2019 | 47.2e | 50.6d | 53.8c | 52.0c | 65.9b | 68.6a | 66.9b | 70.9e | 91.2d | 96.9c | 100.1b | 104b | 113.9a | 116.9a | 98.8e | | 127.2d | | 140.4c | 149.9a | 151.4a | 146.8b | 139.6c |
| Mean | 39.2f | 42e | 45.1d | 48.6c | 54.5b | 59.8a | 59.9a | 76.5f | 89.9e | 98.8d | 107.9c | 117.8b | 130.3a | 127.3a | 102.1d | | 132.6c | | 148.9b | 159.4a | 163.1a | 167.6a | 164.4a |
| ANOVA | | | | | | | | | | | | | | | | | | | | | | | |
| Y | * | | | | | | |  | * | | | | | | |  | | * | | | | | |
| N | * | | | | | | |  | ** | | | | | | |  | | * | | | | | |
| Y*N | * | | | | | | |  | * | | | | | | |  | | * | | | | | |
| Note: Different lowercase letters within a column and different capital letters within a row or column represent significant differences (*P* < 0.05).Means and standard errors of three replicates are presented. | | | | | | | | | | | | | | | | | | | | | | | |
|  |  |  |  |  |  |  |  |  |  |  |  |  |  |  |  |  |  |  |  |  |  |  |  |

| **Table 4S.** Effects of N fertiliser rate on nitrogen accumulation of different organs and N concentration of grain and NHI in dryland Wheat. | | | | | | | | | | | | | | | | | | | | | | | |
| --- | --- | --- | --- | --- | --- | --- | --- | --- | --- | --- | --- | --- | --- | --- | --- | --- | --- | --- | --- | --- | --- | --- | --- |
|  |  |  |  |  |  |  |  |  |  |  |  |  |  |  |  |  |  |  |  |  |  |  |  |
| Year | Leaf (kg ha^–1^) | | | | | | | Stem+sheaths (kg ha^–1^) | | | | | | | Cob+glume (kg ha^–1^) | | | | | | | | |
|  | N0 | N90 | N120 | N150 | N180 | N210 | N240 | N0 | N90 | N120 | N150 | N180 | N210 | N240 | N0 | | N90 | | N120 | N150 | N180 | N210 | N240 |
| 2011-2012 | 0.86d | 0.81d | 1.2b | 1.08c | 1.57a | 1.31b | 1.03c | 15.9e | 17.8d | 18.3c | 19.6b | 21.5a | 20.5a | 19.1b | 0.99e | | 1.58d | | 2.06c | 3.22ab | 3.05b | 3.6a | 3.18ab |
| 2012-2013 | 1.95c | 2.18bc | 3.23a | 1.94c | 2.09c | 2.3b | 2.51b | 16.8d | 22c | 21.4c | 22.4c | 27.4a | 25.9b | 26.4ab | 0.89e | | 1.74d | | 2.71c | 4.28b | 4.29b | 5.34a | 4.57b |
| 2013-2014 | 2.4b | 2.23c | 2.57b | 2.22c | 2.69a | 2.88a | 2.79a | 18.8d | 22b | 20.9c | 23.2ab | 24.9a | 25.1a | 25.5a | 1.02f | | 2.04e | | 2.4d | 3.75c | 3.31c | 5.21a | 4.31b |
| 2014-2015 | 0.84c | 0.92c | 1.24a | 1.17b | 1.39a | 1.11b | 0.93c | 16c | 18.5b | 18.3b | 19.8a | 21a | 20.3a | 19.3a | 1.9e | | 2.74d | | 3.19c | 4.64a | 3.93b | 3.07c | 2.89d |
| 2015-2016 | 1.19b | 0.98c | 1.15b | 0.81c | 1.52a | 1.46a | 1.64a | 17.8c | 22.1b | 21.9b | 22.3b | 26.8a | 25.3ab | 27.2a | 1.01g | | 2.16f | | 2.59e | 3.61c | 3.82c | 4.76b | 5.38a |
| 2016-2017 | 1.7e | 1.65e | 2.04d | 2.78c | 3.77a | 3.14b | 2.92c | 17.5f | 22.6e | 22.5e | 25.2d | 31.8a | 29.1c | 30.4b | 1.16f | | 1.9e | | 2.79d | 4.63b | 4.23c | 5.29a | 4.87b |
| 2017-2018 | 0.8d | 0.94c | 1.39b | 1.34b | 1.46a | 1.49a | 0.99c | 15.9c | 18.3b | 19b | 20.3a | 21.5a | 20.5a | 18.9b | 0.95e | | 1.82d | | 2.28c | 3.53a | 2.95b | 3.68a | 2.72b |
| 2018-2019 | 0.95d | 1.25c | 1.55b | 1.48b | 2.1a | 2.09a | 1.11c | 16.9d | 20c | 19.6c | 21b | 23.6a | 23.4a | 21.9b | 1.17f | | 2.06e | | 2.57d | 3.98b | 3.92b | 5.24a | 3.67c |
| Mean | 1.33c | 1.37c | 1.79b | 1.6b | 2.07a | 1.97a | 1.74b | 16.9d | 20.4c | 20.2c | 21.7b | 24.8a | 25.7a | 23.5a | 1.13f | | 2.0e | | 2.57d | 3.95c | 3.68c | 4.52a | 3.94b |
| Year | Grain (kg ha^–1^) | | | | | | | N concentration of grain (g kg^-1^) | | | | | | | Nitrogen harvest index (NHI) | | | | | | | | |
|  | N0 | N90 | N120 | N150 | N180 | N210 | N240 | N0 | N90 | N120 | N150 | N180 | N210 | N240 | N0 | | N90 | | N120 | N150 | N180 | N210 | N240 |
| 2011-2012 | 54.1d | 74.8c | 86.3b | 98.1a | 97.1a | 98.6a | 95.6a | 23.52a | 22.1c | 22.1c | 22.5b | 22.7b | 23.2a | 22.6ab | 0.64b | | 0.67ab | | 0.68a | 0.69a | 0.71a | 0.67ab | 0.67ab |
| 2012-2013 | 81.4d | 108.9c | 121.8b | 116.1b | 126.1a | 124.7a | 124.5a | 23.2a | 22.5b | 22.2d | 21.2d | 21.9c | 22.7b | 22.7b | 0.68a | | 0.68a | | 0.69a | 0.68a | 0.66b | 0.62c | 0.62c |
| 2013-2014 | 82.1e | 97.1d | 112.3c | 120.9b | 122.9b | 131.1a | 127.6ab | 23.1a | 22.2d | 21.4d | 21.9c | 21.9c | 22.6b | 22.6b | 0.67b | | 0.66b | | 0.68ab | 0.69a | 0.67b | 0.7a | 0.7a |
| 2014-2015 | 58.4e | 78.7d | 87.2c | 100.1a | 97.1a | 96.7ab | 94.9b | 23.6a | 23.3a | 21.7c | 22.2b | 22.7b | 23.2ab | 23.1ab | 0.62c | | 0.65b | | 0.67ab | 0.68a | 0.66ab | 0.64b | 0.67ab |
| 2015-2016 | 67.7d | 95.8c | 108.8b | 114.2a | 117.1a | 117.8a | 117.1a | 23.5a | 22.2b | 21.7c | 21.7c | 22.5b | 22.8ab | 23.1a | 0.66ab | | 0.67b | | 0.68a | 0.68a | 0.67b | 0.66ab | 0.65b |
| 2016-2017 | 78.1d | 102.7c | 118.9b | 123.1a | 125.4a | 122.3a | 123.4a | 24.4a | 22.8c | 22.2c | 22.5c | 22.5c | 23.1b | 23.2b | 0.68a | | 0.68a | | 0.69a | 0.67a | 0.64ab | 0.62b | 0.64ab |
| 2017-2018 | 54.6d | 70.5c | 85.1b | 97.9a | 95.2a | 95.1a | 96.9a | 23.4a | 22.2b | 21.3c | 21.1c | 21.1c | 21.6c | 22.1b | 0.65b | | 0.66ab | | 0.67b | 0.68a | 0.71a | 0.66ab | 0.67b |
| 2018-2019 | 66.1d | 85.8c | 96.3b | 102.6a | 99.5a | 102.1a | 98.8a | 23.2a | 22.2b | 21.7b | 21.1c | 20.9c | 21.9b | 21.9b | 0.66b | | 0.67b | | 0.68a | 0.68a | 0.65b | 0.69a | 0.7a |
| Mean | 67.8d | 89.3c | 102.1b | 109.1a | 110.1a | 111.1a | 109.8a | 23.5a | 22.5b | 21.8c | 21.8c | 22.1c | 22.6b | 22.6b | 0.66b | | 0.67ab | | 0.68a | 0.68a | 0.67ab | 0.66b | 0.67ab |
| ANOVA | | | | | | | | | | | | | | | | | | | | | | | |
| Y | */* | | | | | | |  | */* | | | | | | |  | | */* | | | | | |
| N | */** | | | | | | |  | **/* | | | | | | |  | | */ns | | | | | |
| Y*N | */* | | | | | | |  | */ns | | | | | | |  | | */ns | | | | | |
| Note: Different lowercase letters within a column and different capital letters within a row or column represent significant differences (*P* < 0.05). Means and standard errors of three replicates are presented. | | | | | | | | | | | | | | | | | | | | | | | |
|  |  |  |  |  |  |  |  |  |  |  |  |  |  |  |  |  |  |  |  |  |  |  |  |

| Table 5S**.** Effect of N fertiliser rate on translocation of Pre-/Post anthesis accumulated nitrogen to grain in dryland winter wheat. | | | | | | | | | | | | | | | | | | | | | | | | |
| --- | --- | --- | --- | --- | --- | --- | --- | --- | --- | --- | --- | --- | --- | --- | --- | --- | --- | --- | --- | --- | --- | --- | --- | --- |
|  |  |  |  |  |  |  |  |  |  |  |  |  |  |  |  |  |  |  |  |  |  |  |  |  |
| Year | Translocation of Pre-anthesis accumulated nitrogen to grain (*Wn*) | | | | | | | Translocation of Post-anthesis accumulated nitrogen to grain (*Tn*) | | | | | | | | Rate of translocation from Pre-anthesis accumulated nitrogen to grain (*Wn %*) | | | | | | | | |
|  | N0 | N90 | N120 | N150 | N180 | N210 | N240 | N0 | | N90 | N120 | N150 | N180 | N210 | N240 | N0 | | | N90 | N120 | N150 | N180 | N210 | N240 |
| 2011-2012 | 32.3e | 31.9e | 39.7d | 42.4c | 57.7a | 46.4b | 44.3c | 21.7d | | 42.9b | 46.6b | 55.7a | 39.3c | 52.2a | 51.3ab | 59.8a | | | 52.6c | 56.1b | 57.2b | 59.4a | 57.1b | 56.3b |
| 2012-2013 | 47.7e | 50.1e | 66.3d | 77.8c | 85.4b | 94.8a | 89.4b | 33.7c | | 58.8a | 55.5a | 38.3d | 40.6b | 29.9e | 35.1c | 58.6d | | | 46.1e | 54.4d | 67.1c | 67.7c | 76.1a | 71.8b |
| 2013-2014 | 48.8e | 58.4d | 62.4c | 76.2b | 77.8b | 101.7a | 97.4a | 33.2c | | 38.7c | 49.9a | 44.7b | 45.1b | 29.3d | 30.2d | 59.5c | | | 60.1c | 55.5d | 63.1b | 63.3b | 77.6a | 76.3a |
| 2014-2015 | 39.1d | 40.4c | 44.1c | 53.1b | 52.3b | 62.1a | 65.7a | 19.3e | | 38.3c | 43.2b | 47.1a | 44.7ab | 34.6c | 29.2d | 67.1a | | | 51.3d | 50.4d | 53.1c | 53.9c | 64.2b | 69.2a |
| 2015-2016 | 51.7c | 50.4c | 51.4c | 49.6c | 67.7b | 82.7a | 76.1a | 16.1e | | 45.4b | 57.4a | 64.6a | 49.3b | 35.1d | 40.9c | 76.3a | | | 52.6c | 57.2d | 58.4d | 57.8c | 70.2a | 65.1b |
| 2016-2017 | 49.2e | 58.1d | 62.9cd | 68.7c | 75.1b | 81.3b | 87.8a | 28.8e | | 44.7c | 56a | 54.4a | 50.4b | 41c | 35.6d | 63.1b | | | 56.4c | 52.9c | 55.8c | 59.8b | 66.4b | 71.1a |
| 2017-2018 | 31.1f | 33.7e | 36.3d | 40.3c | 50.9a | 51.5a | 45.2b | 23.5e | | 36.8d | 48.8b | 57.6a | 44.3c | 43.5c | 51.7ab | 57.1c | | | 67.8a | 62.6b | 61.2b | 63.4a | 64.2a | 66.6a |
| 2018-2019 | 38.2d | 49.8c | 52.8c | 52.8c | 52.1c | 69.2b | 76.1a | 27.9e | | 36c | 43.5b | 49.8a | 47.4a | 32.9c | 22.7d | 57.8c | | | 58.1c | 54.8cd | 51.4d | 52.3d | 67.7b | 77.1a |
| Mean | 42.2f | 46.5e | 51.9d | 57.6c | 64.8b | 73.7a | 72.7a | 25.6d | | 42.7b | 50.1a | 51.5a | 45.2b | 37.3c | 37.1c | 62.2b | | | 62.1b | 60.9b | 65.7b | 68.9a | 66.3a | 66.1a |
| ANOVA | | | | | | | | | | | | | | | | | | | | | | | | |
| Y | * | | | | | | |  | * | | | | | | | |  | * | | | | | | |
| N | * | | | | | | |  | ** | | | | | | | |  | * | | | | | | |
| Y*N | * | | | | | | |  | * | | | | | | | |  | * | | | | | | |
| Note: Different lowercase letters within a column and different capital letters within a row or column represent significant differences (*P* < 0.05).Means and standard errors of three replicates are presented. | | | | | | | | | | | | | | | | | | | | | | | | |
|  |  |  |  |  |  |  |  |  |  |  |  |  |  |  |  |  |  |  |  |  |  |  |  |  |
